# Psychological and Social Challenges Parents of Children With Strabismus Face in Qassim Region, Saudi Arabia: A Cross-Sectional Study

**DOI:** 10.7759/cureus.36920

**Published:** 2023-03-30

**Authors:** Saad Althiabi, Abdulrahman AlDarrab, Saad H Alenezi, Basil A Alharbi, Abdulaziz J Aljbreen, Ghaida F Alsalamah, Rahaf A Alotaibi, Futun A Almutairi, Naif A Albadrani

**Affiliations:** 1 Ophthalmology, Unaizah College of Medicine, Qassim, SAU; 2 Surgery/Ophthalmology, Prince Sattam Bin Abdulaziz University, AlKharj, SAU; 3 Department of Ophthalmology, Majmaah University, Al-Majmaah, SAU; 4 Ophthalmology, Ar Rass General Hospital, Burydah, SAU; 5 Ophthalmology, King Saud Hospital, Qassim, SAU; 6 General Practice, Qassim University, Qassim, SAU

**Keywords:** esotropia, parents, children, psychological challenges, strabismus

## Abstract

Aim

Strabismus or squint is when one eye is misaligned or looking in a different direction. Some people consider strabismus as a cosmetic problem, causing patients in this condition to be mishandled, especially children, which might affect their vision and their quality of life. This study aimed to evaluate the psychological and social challenges faced by parents of children with strabismus during the years 2020-2021.

Subjects and methods

This is a cross-sectional study conducted among parents who have children with strabismus onset from birth till the age of 16 years. A self-administered questionnaire was distributed among parents during their visits to the vision eye specialist center. The questionnaire included socio-demographic characteristics of patients with strabismus and a 12-item questionnaire to measure the psychological impact on the parents of children with strabismus.

Results

Out of the 105 respondents, 65.7% were fathers, and 55.2% had children aged six years old or less. The most prominent strabismus type was esotropia (where the eye turns inward; 38.1%), while the most common nature of strabismus was intermittent (76.2%). The mean total score of psychological impact was 36.2 (SD=8.15) out of 60 points, with 62.9% considered as having an average psychological impact. Moreover, 77.1% of the respondents believed that strabismus could be treated and improved.

Conclusion

There was a moderate psychosocial challenge among parents of children with strabismus. Out of all the subjects, mothers who had prior visits to government hospitals were noticed to be greatly affected psychologically as compared to other subjects.

## Introduction

Strabismus is characterized by one eye looking straight ahead while the other eye may turn out, in, down, or up. The misalignment may shift from one eye to the other. In childhood, strabismus may impact vision, and it is a risk factor for the development of amblyopia [1].

Several studies report psychological impact and social bias towards children with strabismus [2,3]. A study conducted in India observed that 96% (n= 120) of parents of children with strabismus had a psychological and social impact because of their child's condition, and 91% of them even considered strabismus as a cosmetic stigma [4]. Another study conducted in Saudi Arabia found that 69% (n=233) of parents and children preferred orthotropic (normal eyes) faces as playmates over strabismic faces [5]. 

In the United States (US), strabismus is present in 2% of African Americans but is found in 3% of White preschool children [6]. However, in a study conducted in Riyadh, the capital city of Saudi Arabia, the prevalence of strabismus was found to be 0.5% in preschool children [7]. In the Qassim region in Saudi Arabia, strabismus is found in almost 5% of primary school children with amblyopia [8]. There could be 450 children with strabismus in the Qassim region among the 85,000 children who are younger than five years [9].

Previous knowledge about strabismus plays a significant role in the early intervention and treatment of strabismus in order to decrease the visual damage and economic burdens to society [10]. Citizens of the Qassim region have good knowledge and an excellent attitude towards strabismus [9]. However, in the Qassim region, no studies have been performed with the aim of understanding the psychological and social impact of strabismus on the parents of children with strabismus. In this study, we aimed to examine the challenges facing parents of children diagnosed with strabismus in the Qassim region of Saudi Arabia.

## Materials and methods

The psychological and social challenges have been assessed using a 12-item questionnaire validated internationally. Each question has a five-point Likert scale category ranging from "strongly disagree", coded as 1, to "strongly agree", coded as 5. The total psychological impact score has been calculated by adding all 12 items, and a score range from 12 to 60 points has been generated, indicating that the higher the score, the higher the psychological impact among the respondents. By using 50% and 75% as cutoff points to classify the psychological impact levels, participants were grouped as having a low impact if the score was less than 50%, as an average level if the score was from 50% to 75%, and as a high impact level if the score was above 75%. 

Descriptive statistics were used to describe the overall group of respondents, including numbers and percentages (categorical variables), mean, and standard deviation (continuous variables). The differences in the score of psychological impact among the socio-demographic and the disease characteristics were conducted by using the Mann-Whitney Z-test. Statistical collinearity was measured using the Shapiro-Wilk test as well as the Kolmogorov-Smirnov test. The psychological impact score follows the non-normal distribution. Therefore, the non-parametric tests were applied. Two-tailed analysis with p<0.05 was used as the cutoff for statistical significance. All data analyses were performed using the Statistical Package for Social Sciences, version 26 (IBM, Inc., Armonk, USA).

## Results

A total of 105 parents filled out the survey. Table 1 presents the socio-demographic characteristics of children and caregivers. Children aged six years or less were 55.2%, and nearly 60% were males. Approximately two-thirds (65.7%) who filled out the questionnaire were fathers. Regarding the mother’s education, 71.4% had a bachelor's degree. Regarding the father's education, 47.6% had a bachelor's degree. In addition, 62.9% reported having children six years old or less since being diagnosed with strabismus.

**Table 1:** Socio-demographic characteristics of the children and caregivers (n=105)

Study data	n (%)
Age group	
≤6 years	58 (55.2%)
>6 years	47 (44.8%)
Gender of the child	
Male	62 (59.0%)
Female	43 (41.0%)
Relationship of the responder with the child	
Mother	32 (31%)
Father	69 (66%)
Other	3 (3%)
Mother's education level	
Intermediate certificate or less	6 (5.7%)
High school diploma	16 (15.2%)
Diploma	3 (2.9%)
Bachelor's degree	75 (71.4%)
Master’s degree	5 (4.8%)
Father's education level	
Intermediate certificate or less	4 (3.8%)
High school diploma	25 (23.8%)
Diploma	10 (9.5%)
Bachelor's degree	50 (47.6%)
Master’s degree	16 (15.2%)
Age diagnosed in years	
≤6 years	66 (62.9%)
>6 years	39 (37.1%)

Table 2 shows that about one-third of the patients were seen for the first time by the specialized clinic in treating strabismus. Nearly 80% visited private clinics or dispensary clinics during their last visit. The most common strabismus type was esotropia (eye turns inward; 38.1%), while intermittent was the most prominent nature of strabismus (76.2%). Approximately 45% showed partial improvement when wearing glasses. According to their families, 61.9% of mothers noticed the presence of strabismus in their children. It is interesting to know that 77.1% of the respondents believed that strabismus could be corrected and improved.

**Table 2:** Characteristics of the patients with strabismus (n=105) *It is either the family did not understand the question, mostly they mean it is alternative or other types of strabismus

Characteristics	n (%)
Was your last visit, the first visit to the pediatric eye and strabismus clinic?	
Yes	37 (35.2%)
No	68 (64.8%)
If the answer is no, did you visit the clinic before? (n=68)	
General clinic	2 (2.9%)
General ophthalmology clinic	13 (19.1%)
Pediatric ophthalmology and strabismus clinic	51 (75%)
Other	2 (3%)
Your last visit was in?	
Government hospital	22 (21.0%)
Dispensary or private clinic	83 (79.0%)
Strabismus type	
Esotropia (eye turns inward)	40 (38.1%)
Exotropia (eye turns outward)	23 (21.9%)
Hypertropia	7 (6.7%)
Other*	35 (33.3%)
The nature of strabismus	
Permanent	25 (23.8%)
Intermittent	80 (76.2%)
Does your child get better when he wears glasses?	
Complete improvement	34 (32.4%)
Partial improvement	47 (44.8%)
Not getting better	24 (22.9%)
Before the diagnosis, who noticed the presence of strabismus in your child?	
Mother	65 (61.9%)
Father	23 (21.9%)
One of the relatives	11 (10.5%)
Others	6 (5.7%)
Do you think that strabismus is treatable and improving?	
Yes	81 (77.1%)
No	1 (1%)
Probably	23 (21.9%)

Table 3 shows the assessment of the psychological impact on parents and children. It can be observed that the top three highest-rated statements that gave an impact psychologically were "You feel that your child's squint is bothering you" (mean score of 4.36), "You feel that others often notice that your child has a squint when he or she interacts with them" (mean score of 3.70), and "You feel that your child's injury may affect his or her performance" (mean score of 3.43). The overall mean psychological impact score was 36.2 (SD=8.15) with 62.9% categorized as having an average impact, 17.1% categorized as high, and 20% categorized as having low psychological impact levels.

**Table 3:** Assessment of psychological impact among parents and children (n=105) Responses ranged from "strongly disagree", coded as 1, to "strongly agree", coded as 5.

How much do you agree with the following statement	Mean ± SD
You feel that your child's squint is bothering you	4.36 ± 0.79
You feel that your child's injury may affect his or her performance	3.43 ± 1.35
You feel that others often notice that your child has a squint when he or she interacts with them	3.70 ± 1.07
You feel that your child's strabismus limits his vision	3.34 ± 1.26
You feel that having a squint is a cosmetic defect	3.14 ± 1.28
You feel that your child is showing signs of psychological impact because of his squint	2.84 ± 1.22
You feel that asking someone about your child's condition upsets you	2.79 ± 1.29
You feel that your child is finding it difficult to read because of his squint	2.75 ± 0.96
You feel that some people look long at your child because of his strabismus	2.66 ± 1.09
You feel that your child's squint may limit his chances of building social relationships	2.63 ± 1.21
You feel that some people ignore your child because he has a squint	2.30 ± 0.97
You feel that your child is finding it difficult to find friends because of his squint	2.20 ± 1.13
Total psychological impact score	36.2 ± 8.15
Level of psychological impact	(n, %)
Low	21 (20.0%)
Average	66 (62.9%)
High	18 (17.1%)

When measuring the differences in the score of psychological impact in relation to the socio-demographic characteristics of respondents (Table 4), it was found that a higher psychological impact score was more associated with being a mother to a child (Z=2.833; p=0.005) and the last visit to a governmental hospital (Z=2.879; p=0.004). Other socio-demographic variables did not show significant differences with the overall psychological impact score (p>0.05).

**Table 4:** Differences in the psychological impact score in relation to the socio-demographic characteristics of the children with strabismus and caregivers (n=105) ^§^ p-value has been calculated using Mann Whitney Z-test. ** Significant at p<0.05 level.

Factor	Psychological score (max=60); mean ± SD	Z-test	p-value^§^
Age group			
≤6 years	36.9 ± 8.16	0.694	0.488
>6 years	35.3 ± 8.15		
Gender of the child			
Male	36.6 ± 8.14	1.067	0.286
Female	35.5 ± 8.22		
Relationship with the child			
Mother	39.7 ± 8.53	2.833	0.005**
Father	34.3 ± 7.36		
Mother's education level			
Diploma or below	36.2 ± 4.78	0.26	0.795
Bachelor's or higher	36.1 ± 8.98		
Father's education level			
Diploma or below	36.8 ± 7.08	0.757	0.449
Bachelor's or higher	35.8 ± 8.76		
Age diagnosed in years			
<5 years	35.8 ± 8.59	0.744	0.457
≥5 years	36.7 ± 7.44		
Was your last visit, the first visit to the pediatric eye and strabismus clinic?			
Yes	34.7 ± 9.30	1.834	0.067
No	36.9 ± 7.41		
Type of hospital during the last visit			
Government hospital	40.6 ± 8.13	2.879	0.004**
Dispensary or private clinic	34.9 ± 7.78		
Strabismus is treatable and improving			
Yes	36.4 ± 8.87	0.42	0.674
No/Probably	35.4 ± 5.09		

## Discussion

The present study investigated the psychological and social challenges dealing with parents of strabismic children. In this study, we measured the overall psychological impact on parents by using a 12-items questionnaire. Based on the results, the overall psychological impact score was 36.2 (SD=8.15) out of 60 points. According to the given criteria, the majority (62.9%) were categorized as having an average psychological impact, 17.1% were categorized as high, and 20% were categorized as having a low impact level. Furthermore, increased psychological challenges can be significantly predicted among mothers and those who had the last visit to a government hospital. The negative impact of children with strabismus among parents was also discussed in a study done by Schuster et al. [11]. According to their reports, children and adolescents with strabismus were noticed to have a lower health-related quality of life in terms of mental health problems like hyperactivity/inattention and peer problems that greatly affected their parents. In another study done by Akay et al. [12], they compared the psychological disorders of mothers with strabismic versus healthy children. They found out that the mothers of children with strabismus had significantly higher depression compared to the control group mothers. They also exhibited significantly lower scores in democratic attitude, meaning they failed to establish a supportive and friendly relationship with their kids. These mothers were also associated with rejection of the maternal role more than those mothers in the control group. In contrast to this scenario, Alzuhairy et al. [9] documented that the parents of children with strabismus showed excellent knowledge about the signs and symptoms of the disease (50.6%) and also had a positive attitude (70.4%). Parents who have a good understanding of strabismus may have better management which could lead to a better psychological health condition.

The top five most common psychological challenges facing the parents were related to bothersome child squint, feeling that child squint may affect performance, the belief that others may notice their children squint upon interacting with others, feeling that strabismus may limit the child's vision, and belief that squint is a cosmetic deficiency. Consistent with our reports, these psychological difficulties being confronted by our population had also been mentioned and had been a challenge among the Indian population. According to the study done by Singh et al. [4], nearly all parents (96.7%) were bothered by the strabismus of their children, 91.2% considered squint a cosmetic stigma, 84.2% felt that their child's strabismus was observed by their peers during the interaction, 61.7% felt that making friends might be difficult for their children, and three-quarters (75%) felt unpleasant if someone asked something about their child's strabismus. The study concluded that some parents, specifically the less educated ones, showed a lack of understanding of strabismus, leading to a late diagnosis and ineffective countermeasures toward the disease. Contradicting these reports, a study carried out among Indian parents in urban areas [13] found that many of the parents (53.3%) were not concerned about what people think about their children's eyes, and nearly half of them (47.7%) never thought that people noticed their child's eyes unless they said something about it. 

Moreover, some of the general characteristics of children with strabismus could contribute to parents' psychological condition. For instance, nearly two-thirds (64.8%) of the parents did not visit a pediatric eye or strabismus clinic during their last or first visit, while visiting the right specialist is vital to address the issue. We also discovered that the most commonly reported strabismus type was esotropia (where the eye turns inward; 38.1%), while intermittent was the most prominent nature of strabismus (76.2%). Approximately one-quarter of parents (22.9%) believed that their child did not improve when wearing eyeglasses. While our respondents were affected by these incidents, in Jeddah [10], most respondents found strabismus to be treatable (71.5%) and had a good understanding of the risk factors and complications of strabismus. Family history (16%) and refractive eye errors (12.9%) were the most reported risk factors for developing strabismus. In addition, subjects agreed that if strabismus is left untreated, complications might occur, such as visual loss, cosmetic stigma, and poor self-image (55.2%).

Believing that strabismus can be treated and improved likely had a positive effect on parents with strabismic children. In our study, 77.1% of our parents agreed that strabismus is treatable. Only 1% of parents did not think the strabismus can be improved; this small portion of subjects can be explained by the setting of our study because parents who seek medical advice think their child's strabismus will be treatable. In Switzerland [14], surgical intervention for strabismus has been raised as the most probable treatment solution for young children. According to their reports, the acceptance of peers for children aged six years or older with a visible squint seems to be unlikely. Hence, since this pessimistic attitude towards strabismus appears to emerge at approximately the age of six years, therefore, corrective surgery for strabismus without prospects for binocular vision should be carried out before this age. Similarly, strabismus surgery could result in positivity in the quality of life among children. Temeltürk et al. [15] noted that children with strabismus had significantly fewer psychological issues and had a better quality of life post-surgery. Also, they were seen to have decreased anxiety symptom levels, including their parents, and the rates of ocular realignment were positively correlated with improvement in social and attention problems among children.

Limitations

One of the major limitations of this study is related to the sample size. Our sample was relatively small (n=105). It could be interesting to see a bigger sample that could generate better results which could provide a more accurate view of the psychological and social challenges facing parents of children with strabismus. Also, cross-sectional study, in its nature, is prone to disadvantages, including cause-and-effect relationships and bias. 

## Conclusions

There was a moderate psychosocial challenge among parents of children with strabismus. Of all the subjects, mothers who had prior visits to government hospitals were seen to be greatly affected psychologically as compared to other subjects. Furthermore, bothersome child's squint, worries that squint may affect child performance, limiting child's vision, and being noticed by others were some of the factors affecting the psychological state of the parents. The psychological effect of this disease can be decreased by health education targeting parents of children suffering from strabismus. Addressing psychological challenges experienced by parents could lead to better disease management which eventually will improve both parents and children's quality of life.

## Appendices

Questionnaire

Do you agree to participate in this survey?

•  Yes

•  No

Who is the participant?

•  Mother

•  Father

•  Other

Level of mother education:

•  Elementary certificate OR less

•  Secondary certificate

•  Diploma certificate

•  Bachelor’s certificate

•  Doctorate

Level of father education:

•  Elementary certificate OR less

•  Secondary certificate

•  Diploma certificate

•  Bachelor’s certificate

•  Doctorate

Child’s gender:

•  Male

•  Female

Child’s age ‘now’:

…………

Child’s age when diagnosed with strabismus:

…………

**Was your last visit the first visit to a pediatric ophthalmology and strabismus clinic? **           

•  Yes

•  No

If not, what is the clinic you visited previously?

•  General clinic

•  General ophthalmology clinic

•  Pediatric ophthalmology and strabismus Clinic

•  Other

•  First visit

Your last visit was to

•   Public hospital

•   Private clinic or center

Strabismus type:

•  Eye turns inward (Esotropia)

•  Eye turns outward (Exotropia)

•  Hypertropia/Hypotropia

•  Other

Strabismus nature:

•  Constant

•  Intermittent (or transient)

Does your child improve when (s)he is wearing glasses?

•  Complete improvement

•  Partial improvement

•  No improvement

Before the diagnosis, who noticed the presence of strabismus in your child?

•   Mother

•   Father

•   Relatives

•   Others

How much do you agree with the following statements?

You feel that your child's strabismus bothers you

•    strongly agree

•    agree

•    neutral

•    disagree

•    strongly disagree

You feel that other people often notice your child's strabismus when he deals with them

•    strongly agree

•    agree

•    neutral

•    disagree

•    strongly disagree

You feel that your child's strabismus may affect his or her performance (at school or at work)

•    strongly agree

•    agree

•    neutral

•    disagree

•    strongly disagree

You feel that your child's strabismus might limit the chances of your child to build social relationships

•    strongly agree

•    agree

•    neutral

•    disagree

•    strongly disagree

You feel that your child has difficulty in finding friends due to his strabismus

•    strongly agree

•    agree

•    neutral

•    disagree

•    strongly disagree

You feel annoyed when someone asks you about your child's condition

•    strongly agree

•    agree

•    neutral

•    disagree

•    strongly disagree

You feel that some people avoid looking at your child because of his/her strabismus

•    strongly agree

•    agree

•    neutral

•    disagree

•    strongly disagree

You feel some people stare for a long time at your child due to their strabismus

•    strongly agree

•    agree

•    neutral

•    disagree

•    strongly disagree

You feel that strabismus decreases your child's vision

•    strongly agree

•    agree

•    neutral

•    disagree

•    strongly disagree

Your child has difficulty in reading because of strabismus

•    strongly agree

•    agree

•    neutral

•    disagree

•    strongly disagree

Your child shows signs of being psychologically affected by strabismus

•    strongly agree

•    agree

•    neutral

•    disagree

•    strongly disagree

You think that squint is a cosmetic stigma

•    strongly agree

•    agree

•    neutral

•    disagree

•    strongly disagree

You think that strabismus is treatable

•   yes 

•   maybe 

•   no 
